# Supplementary material for: Post-acute care for frail older people decreases 90-day emergency room visits, readmissions and mortality: An interventional study
Source: PLoS One. 2023 Jan 6;18(1):e0279654. doi: 10.1371/journal.pone.0279654 (PMC9821781; doi:10.1371/journal.pone.0279654)
Supplement: S1 Table — (DOCX) [file pone.0279654.s002.docx]

**S1 Table. Univariate analysis of the factors associated with emergency room visits within 90 days (n=254)**

| Variables | Odds ratio | 95% CI | *p* value |
| --- | --- | --- | --- |
| PAC | 0.48 | 0.25-0.91 | 0.023 |
| Duration of PAC (≧15 days) | 0.20 | 0.10-0.41 | <0.001 |
| Gender (women) | 0.74 | 0.43-1.28 | 0.28 |
| Age ≧ 85 (year) | 0.67 | 0.38-1.18 | 0.17 |
| Dementia | 0.49 | 0.26-0.91 | 0.023 |
| Parkinsonism | 0.92 | 0.37-2.31 | 0.86 |
| Chronic kidney disease | 1.33 | 0.77-2.29 | 0.30 |
| Chronic obstructive pulmonary disease | 1.84 | 0.91-3.72 | 0.09 |
| Main caregiver (Family) | 0.83 | 0.48-1.44 | 0.50 |
| Living in second floor (or above) | 1.71 | 0.85-3.46 | 0.13 |
| Baseline condition | | | |
| ADL dependence | 0.88 | 0.39-2.00 | 0.76 |
| IADL dependence | 0.39 | 0.05-3.29 | 0.68 |
| Severe frailty (CFS) | 1.54 | 0.90-2.65 | 0.12 |
| High fall risk (STEADI) | 0.68 | 0.39-1.19 | 0.17 |
| Severe cognitive impairment (SPMSQ)^1^ | 1.83 | 0.87-3.84 | 0.11 |
| Depression (GDS) | 1.17 | 0.65-2.12 | 0.60 |
| Delirium (CAM) | 0.52 | 0.28-0.96 | 0.036 |
| Malnutrition (MNA)^2^ | 1.10 | 0.49-2.45 | 0.82 |
| Severe problem in mobility (EQ-5D) | 1.48 | 0.85-2.57 | 0.17 |
| Severe problem in self-care (EQ-5D) | 2.04 | 1.18-3.54 | 0.010 |
| Severe problem in usual activities (EQ-5D) | 2.23 | 1.29-3.86 | 0.004 |
| Severe pain / discomfort (EQ-5D) | 1.81 | 0.77-4.29 | 0.17 |
| Severe anxiety / depression (EQ-5D) | 2.35 | 0.95-5.80 | 0.06 |

^1^ SPMSQ score **≧ 8.**

^2^ Malnutrition: MNA score < 12.

Abbreviations: ADL, activities of daily living; CAM, Confusion Assessment Method; CFS, Clinical Frailty Scale; EQ-5D, EuroQol-5 dimension; GDS, Geriatric Depression Scale; IADL, instrumental activities of daily living; MNA, Mini Nutrition Assessment; PAC, post-acute care; SPMSQ, Short Portable Mental Status Questionnaire; STEADI, Stop Elderly Accidents, Deaths, and Injuries.
