# Supplementary material for: Post-acute care for frail older people decreases 90-day emergency room visits, readmissions and mortality: An interventional study
Source: PLoS One. 2023 Jan 6;18(1):e0279654. doi: 10.1371/journal.pone.0279654 (PMC9821781; doi:10.1371/journal.pone.0279654)
Supplement: S2 Table — (DOCX) [file pone.0279654.s003.docx]

**S2 Table. Univariate analysis of the factors associated with readmission within 90 days (n=254)** .

| Variables | Odds ratio | 95% CI | *p* value |
| --- | --- | --- | --- |
| PAC | 0.82 | 0.43-1.56 | 0.54 |
| Duration of PAC (≧15 days) | 0.30 | 0.17-0.56 | <0.001 |
| Gender (women) | 0.88 | 0.53-1.48 | 0.64 |
| Age ≧ 85 (year) | 0.83 | 0.48-1.44 | 0.51 |
| Dementia | 0.83 | 0.47-1.44 | 0.50 |
| Parkinsonism | 0.23 | 0.07-0.78 | 0.011 |
| Chronic kidney disease | 1.31 | 0.78-2.21 | 0.30 |
| Chronic obstructive pulmonary disease | 1.74 | 0.87-3.47 | 0.11 |
| Main caregiver (Family) | 1.45 | 0.84-2.49 | 0.18 |
| Living in second floor (or above) | 1.18 | 0.63-2.22 | 0.61 |
| Baseline condition | | | |
| ADL dependence | 0.66 | 0.29-1.49 | 0.32 |
| IADL dependence | 0.30 | 0.04-2.54 | 0.43 |
| Severe frailty (CFS) | 2.00 | 1.18-3.37 | 0.009 |
| High fall risk (STEADI) | 0.87 | 0.51-1.49 | 0.62 |
| Severe cognitive impairment (SPMSQ)^1^ | 1.35 | 0.65-2.83 | 0.42 |
| Depression (GDS) | 1.16 | 0.66-2.05 | 0.61 |
| Delirium (CAM) | 0.60 | 0.34-1.06 | 0.08 |
| Malnutrition (MNA)^2^ | 0.82 | 0.37-1.83 | 0.63 |
| Severe problem in mobility (EQ-5D) | 1.65 | 0.97-2.80 | 0.06 |
| Severe problem in self-care (EQ-5D) | 1.99 | 1.18-3.37 | 0.010 |
| Severe problem in usual activities (EQ-5D) | 2.43 | 1.43-4.12 | 0.001 |
| Severe pain / discomfort (EQ-5D) | 1.65 | 0.71-3.85 | 0.24 |
| Severe anxiety / depression (EQ-5D) | 2.19 | 0.89-5.37 | 0.08 |

^1^ SPMSQ score **≧ 8.**

^2^ Malnutrition: MNA score < 12.

Abbreviations: ADL, activities of daily living; CAM, Confusion Assessment Method; CFS, Clinical Frailty Scale; EQ-5D, EuroQol-5 dimension; GDS, Geriatric Depression Scale; IADL, instrumental activities of daily living; MNA, Mini Nutrition Assessment; PAC, post-acute care; SPMSQ, Short Portable Mental Status Questionnaire; STEADI, Stop Elderly Accidents, Deaths, and Injuries.
