# Supplementary material for: Post-acute care for frail older people decreases 90-day emergency room visits, readmissions and mortality: An interventional study
Source: PLoS One. 2023 Jan 6;18(1):e0279654. doi: 10.1371/journal.pone.0279654 (PMC9821781; doi:10.1371/journal.pone.0279654)
Supplement: S3 Table — (DOCX) [file pone.0279654.s004.docx]

**S3 Table. Univariate analysis of the factors associated with mortality within 90 days (n=254)**

| Variables | Odds ratio | 95% CI | *p* value |
| --- | --- | --- | --- |
| PAC | 0.32 | 0.12-0.83 | 0.014 |
| Duration of PAC (≧15 days) | 0.18 | 0.04-0.77 | 0.014 |
| Gender (women) | 0.83 | 0.33-2.08 | 0.70 |
| Age ≧ 85 (year) | 1.89 | 0.61-5.84 | 0.32 |
| Dementia | 1.39 | 0.54-3.53 | 0.49 |
| Parkinsonism | 1.02 | 0.22-4.67 | 1.00 |
| Chronic kidney disease | 0.73 | 0.28-1.91 | 0.53 |
| Chronic obstructive pulmonary disease | 0.97 | 0.27-3.48 | 1.00 |
| Main caregiver (Family) | 0.92 | 0.36-2.34 | 0.86 |
| Living in second floor (or above) | 0.49 | 0.19-1.30 | 0.15 |
| Baseline condition | | | |
| ADL dependence | 1.20 | 0.33-4.34 | 0.73 |
| IADL dependence | - | - | - |
| Severe frailty (CFS) | 2.68 | 0.99-7.20 | 0.044 |
| High fall risk (STEADI) | 0.79 | 0.31-2.02 | 0.63 |
| Severe cognitive impairment (SPMSQ)^1^ | 0.70 | 0.16-3.17 | 1.00 |
| Depression (GDS) | 1.09 | 0.40-2.96 | 0.86 |
| Delirium (CAM) | 0.48 | 0.16-1.51 | 0.31 |
| Malnutrition (MNA)^2^ | 0.76 | 0.17-3.42 | 1.00 |
| Severe problem in mobility (EQ-5D) | 1.49 | 0.59-3.74 | 0.40 |
| Severe problem in self-care (EQ-5D) | 2.44 | 0.96-6.21 | 0.05 |
| Severe problem in usual activities (EQ-5D) | 3.54 | 1.31-9.54 | 0.009 |
| Severe pain / discomfort (EQ-5D) | 0.48 | 0.06-3.78 | 0.70 |
| Severe anxiety / depression (EQ-5D) | 0.56 | 0.07-4.43 | 1.00 |

^1^ SPMSQ score **≧ 8.**

^2^ Malnutrition: MNA score < 12.

Abbreviations: ADL, activities of daily living; CAM, Confusion Assessment Method; CFS, Clinical Frailty Scale; EQ-5D, EuroQol-5 dimension; GDS, Geriatric Depression Scale; IADL, instrumental activities of daily living; MNA, Mini Nutrition Assessment; PAC, post-acute care; SPMSQ, Short Portable Mental Status Questionnaire; STEADI, Stop Elderly Accidents, Deaths, and Injuries.
