# Supplementary material for: Post-acute care for frail older people decreases 90-day emergency room visits, readmissions and mortality: An interventional study
Source: PLoS One. 2023 Jan 6;18(1):e0279654. doi: 10.1371/journal.pone.0279654 (PMC9821781; doi:10.1371/journal.pone.0279654)
Supplement: S1 File — (DOCX) [file pone.0279654.s005.docx]

研究計畫書

版本/日期：Ver1.4_1090618

簽名/日期：

| 中文計畫名稱 | 衰弱老人急性後期居家整合照護計畫 |
| --- | --- |
| 英文計畫名稱 | Home-Based Integrated Post-Acute Care Program for Frail Elderly |
| 申 請 單 位 | 臺北市立聯合醫院 教研醫務長辦公室 |
| 計畫主持人/共同主持人 | 吳岱穎 醫務長 |
| 協同主持人及相關研究人員 | 研究人員：李旻璋 物理治療師、黃柏諭 研究助理 |
| 計 畫 期 限  **(預計開始收案至繳交結案報告期間)** | 西元 2020 年 1 月 1 日起，  西元 2020 年 12 月 31 日迄；  合計 1 年 0 月 0 日 |
| 計 畫 聯 絡 人 | 姓 名： 吳岱穎  電 話：(公) 02-2552-6714  手 機 : 0979-306-238 |
| 通　訊　地　址 | 臺北市大同區鄭州路145號6樓 |

**ㄧ、計畫摘要**

**前言：**因應人口老化，高齡患者在急性醫療後發生失能情況，此徵兆將造成對醫療體系、家庭及社會照顧之負擔或依賴，故需盡早規劃急性後期之醫療整合照護模式(Post-acute Care，以下稱PAC)。

**研究方法：**本研究屬回溯性資料庫分析研究，蒐集在臺北市立聯合醫院接受急性後期整合照護計畫衰弱高齡（Frailty PAC）的個案病歷資料進行分析，從2017年9月到2020年5月。我們將資料庫中的資料分成3組，居家組、住院組與對照組，居家組以及對照組是接受Frailty PAC團隊提供急性後期整合照護，兩組照護時間皆為2-4周。對照組為符合條件但無意願參與者。本計畫成效評估包含功能性表現、衰弱程度、認知功能、憂鬱程度、營養狀況、潛在不當用藥，在服務開始前，與結案時分別進行評估；此外，病人的再入院率、急診率也會納入分析評估。

**預期結果：**我們預期會得知接受Frailty PAC服務後，其各項成效的提升，包含：日常生活功能、衰弱程度、急性瞻妄情形、營養狀態、憂鬱與認知情形、用藥狀態、跌倒改善以及接受Frailty PAC服務3個月內急診率與再住院率的下降等改善狀況。

**二、計畫緣起、目的**

人口老化以及高齡患者在急性醫療後發生失能情況，將大幅造成對醫療體系、家庭及社會照顧之負擔或依賴，故需盡早規劃急性後期之醫療整合照護模式(Post-acute Care，以下稱PAC)。我們預期透過急性後期照護模式，在治療黃金期內立即給予積極性之整合性照護，使其恢復功能，將可減少後續再住院風險、醫療費用、減輕家庭及社會照顧之負擔。

本研究目的旨在探討執行Frailty PAC的成效，包含：日常生活功能、衰弱程度、急性瞻妄情形、營養狀態、憂鬱與認知情形、用藥狀態、跌倒改善以及接受Frailty PAC服務3個月內急診率與再住院率的下降等。

**三、計畫過程與方法**

本研究屬回溯病歷的資料庫分析，分析對象來源為臺北市立聯合醫院接受急性後期整合照護計畫衰弱高齡（Frailty PAC）的患者，必須經醫事人員訪視評估後，達符合服務條件，方能開始接受Frailty PAC服務，個案之資料維護，由特定管理師更新及整理。

為確保受試者隱私和個人資訊安全，研究人員向管理單位申請資料，並提出擬分析的資料欄位之需求，再由該單位管理師將資料去連結，或變更為無法辨識特定個人之資料、檔案、文件、資訊，並將資料串聯好並去連結後，改以數字編碼取代個案的各項身分識別，去除所有可辨認個資的地方，才交予研究人員做後端的資料分析，研究人員並不直接接觸個案。

研究人員將取得之去連結資料進行整理，從2017年9月起至2020年5月底止，在臺北市立聯合醫院接受Frailty PAC服務的患者總人數共約260人，將其分成居家組約80位、住院組約120位以及對照組約60位，居家組以及住院組接受Frailty PAC團隊提供急性後期整合照護，兩組照護時間皆為2-4周，對照組為符合條件但無意願參與者。主要分析目的在描述接受Frailty PAC服務者(居家組及住院組)與對照組間的差異，分析變項包括:

1. 基本資料：年齡、性別、教育程度、居住狀態、主要照顧者、共病症。
2. 住院資料：住院診斷、住院日期、出院日期、住院併發症、手術治療、有無出備會議。
3. 急性後期整合照護：收案日期、結案日期、治療次數、第一次照護服務前和最後一次照護服務後的成效評估。
4. 主要成效評估：
5. 日常生活:巴氏日常生活量表(Barthel Index)、工具性日常生活功能 (IADL)。
6. 衰弱程度:臨床衰弱量表(Clinical Frailty Scale，CFS)。
7. 次要成效評估：
8. 認知功能: SPMSQ。
9. 憂鬱狀態: Geriatric Depression Scale-5 Item (GDS-5) 。
10. 急性瞻妄評估: Confusion Assessment Method (CAM) 。
11. 跌倒風險: STEADI流程評估與介入。
12. 潛在不當用藥: 2015年Beer’s criteria。
13. 營養狀況評估: MNA Short Form。
14. 生活品質評估量表: EQ-5D。
15. 出院後14、30、60、90天急診與再入院次數及原因。

Frailty PAC服務時間約為2周，本研究將針對接受Frailty PAC服務之實驗組及住院組與未接受Frailty PAC服務的對照組比較分析上述主要及次要成效評估資料並撰寫文章，報告分析結果與組間差異。

統計分析使用SPSS(或是SAS)軟體，以描述性統計呈現數據，以平均數±標準差描述連續性變項數據；計次或百分比描述非連續性變項。Frailty PAC介入前、後，以變異數分析（analysis of variance）連續性變項之差異性，以卡方檢定（chi square test）分析非連續性之變項之差異性。P<0.05視為統計上有顯著意義。

本研究預定進度將於計畫通過後:

1. 文獻回顧：於倫理審查委員會通過後第一個月開始執行。
2. 病歷回溯、資料蒐集及整理：於倫理審查委員會通過後第二個月開始執行。
3. 統計分析：於資料整理完後開始執行分析，預計在倫理審查委員會通過後第三個月進行。
4. 報告撰寫及投稿：於倫理審查委員會通過後開始進行，隨著資料蒐集整理、統計分析過程，逐步完成報告，並投稿國際會議與期刊。預計於倫理審查委員會通過後第五到第六個月開始執行。

可能遭遇的困難為資料之彙整、紀錄不完整，預計透過資料篩選以及統計方法校正分析等方式，解決前述困難。

**四、預期效益**

本研究預期將得知接受Frailty PAC服務過後，其各項成效的提升，包含：日常生活功能、衰弱程度、急性瞻妄情形、營養狀態、憂鬱與認知情形、用藥狀態、跌倒改善以及接受Frailty PAC服務3個月內急診率與再住院率的下降等。

| 以Gantt Chart表示 第 1 年 度之執行進度。 | | | | | | | | | | | | | |
| --- | --- | --- | --- | --- | --- | --- | --- | --- | --- | --- | --- | --- | --- |
| 月 次  工作項目 | 第  1  月 | 第  2  月 | 第  3  月 | 第  4  月 | 第  5  月 | 第  6  月 | 第  7  月 | 第  8  月 | 第  9  月 | 第  10  月 | 第  11  月 | 第  12  月 | 備 註 |
| Seeking IRB approval |  |  |  |  |  |  |  |  |  |  |  |  |  |
| Review of literature |  |  |  |  |  |  |  |  |  |  |  |  |  |
| Data collection |  |  |  |  |  |  |  |  |  |  |  |  |  |
| Statistical analysis |  |  |  |  |  |  |  |  |  |  |  |  |  |
| Manuscript preparation and writing |  |  |  |  |  |  |  |  |  |  |  |  |  |
| Manuscript submission |  |  |  |  |  |  |  |  |  |  |  |  |  |
| 計畫進度 | 10 | 20 | 25 | 30 | 40 | 45 | 50 | 60 | 70 | 80 | 90 | 100 |  |

**五、預定進度**

**六、相關文獻**

1. 衛生局；全民健康保險急性後期整合照護計畫；2017/06/26
2. Miquel Angel Mas,Sergi Sabate, Marco Inzitari,Sebastia J Santaeugenia Gonzalez;Hospital-at-home Integrated Care Programme for the management of disabling health crises in older patients:Comparison with bed-based Intermediate Care; Age and Ageing 2017; 0: 1–7
3. American Geriatrics Society 2015 Beers Criteria Update Expert Panel; American Geriatrics Society 2015 Updated Beers Criteria for Potentially Inappropriate Medication Use in Older Adults; J Am Geriatr Soc 63:2227–2246, 2015

**七 研究計畫預算支用表**

| 壹、研發經費 | | | | | | |
| --- | --- | --- | --- | --- | --- | --- |
| 項 目 | | 摘 要 | | 說 明 | | 金 額 |
| 作  業  費 | 耗材費 | 1. 資料整理費 | | 資料整理 | | 10,000 |
|  |  | 1. 印刷費 | | 影印費、印表機耗材 | | 356 |
|  |  | 1. 其他 | | 健保補充費等雜支 | | 191 |
|  | 人事費 | 1. 碩士級專兼任研究助理薪資 | | 訪問病人、查閱核對病歷、整理資料、行政庶務、報帳等。 (含月支費用、年終獎金、勞健保費雇主負擔部分、勞工退休金雇主負擔部分) x 1名 | | 384,453 |
|  | 審查費 | 1. 人體試驗相關審查費 | | 申請醫院倫理審查費 | | 5000 |
|  | 合 計： | | | | | |
| 貳、院外機構提供之配合項目(本項不列入研發經費項內,僅供參考) | | | | | | |
| 配  合  項  目 | 摘 要 | 金 額 | 品 名 | | 備 考 | |
|  |  |  |  | |  | |
|  |  |  |  | |  | |
|  | 合 計 |  | | | | |
|  | | | | | | |
