# Supplementary material for: Post-acute care for frail older people decreases 90-day emergency room visits, readmissions and mortality: An interventional study
Source: PLoS One. 2023 Jan 6;18(1):e0279654. doi: 10.1371/journal.pone.0279654 (PMC9821781; doi:10.1371/journal.pone.0279654)
Supplement: S2 File — (DOCX) [file pone.0279654.s006.docx]

Study Protocol

Version/Date：Ver1.4_1090618

| Title | Home-Based Integrated Post-Acute Care Program for Frail Elderly |
| --- | --- |
| Administration | Taipei City Hospital Zhong-xing branch |
| Principle Investigator | Tai-Yin Wu M.D., PhD. |
| Research assistants | Min-chang Lee PT, M.S.  Po-Yu Huang B.S. |
| Study date | Start from: 2020 / 1 / 1  End at: 2020 / 12 / 31  Total: 1 year 0 month 0 day |
| Contract person | Name: Tai-Yin Wu  Tel: 02-2552-6714  Phone: 0979-306-238 |
| Address | No.145, Zhengzhou Rd., Datong Dist., Taipei City 10341, Taiwan |

1. **Abstract**

**Background:** As aging population, the elderly may suffer from disabling after acute illness. This could have a great burden on their families, medical system, and society. Therefore, there was an urgent need to plan integrated post-acute care (PAC).

**Methods:** This was a retrospective study. We analyzed the medical records of patients who received PAC-frailty in the Taipei City Hospital from September 2017 to May 2020. They were divided into three groups (home-based PAC, hospital-based PAC, and control group). Those in the home-based and hospital-based PAC groups received PAC for two to four weeks. For those who were eligible but had no willingness to participate PAC was considered the control group. Outcome measurements including functional performances, frail status, cognitive function, depression, nutrition status, and medication were assessed before and after PAC. Also, readmissions and emergency room (ER) visits were analyzed.

**Expected outcomes:** We expect that outcomes would be improved including activities of daily living, frail status, acute delirium, nutrition status, depression, medications, and fall risk. The readmissions and ER visits would decrease within 3 months after PAC.

1. **Purpose**

In an aging society, it would be a great burden to families, the medical system, and society that older patients suffer from disabling after acute hospitalization. Therefore, there is an urgent need to plan integrated PAC. We expect that patients would restore their function, and reduce risks of readmission, costs of medical expenditure, and care burden on families and society by giving intensively integrated care within a good recovery period.

This study is aimed to investigate effects of PAC-frailty including daily function, frail status, acute delirium, nutrition status, depression, cognition, medication, and fall risk. The readmissions rates and ER visits after PAC within 3 months were also analyzed.

1. **Method**

This is a retrospective study. We will review the medical records of patients who received PAC-frailty in the Taipei City Hospital. These data were preserved and updated by a PAC case manager. To ensure the privacy of the subjects and personal information, our research assistant will apply to the PAC case manager for the specific data of PAC-frailty which will be analyzed in our study. After de-identification, these data will be available to our assistants who will not have contact with these patients.

The data will be sorted by their date which is from September 2017 to May 2020. The total number of patients who received PAC-frailty in the Taipei City Hospital is about 260 including 80 in the home-based group, 120 in the hospital-based group, and 60 in the control group. Both home-based and hospital-based groups received PAC-frailty service for two to four weeks. For those who were eligible but had no willingness to participate PAC was considered the control group.

This study aims to analyze the differences between the PAC groups and the control group. We collect the following variables:

1. Demographic variables: age, gender, education level, living status, primary caregiver, and comorbidities.
2. Clinical variables: index diagnosis, date of admission, date of discharge, complications, surgery, and whether there is a family meeting during hospitalization.
3. PAC variables: date of admission, date of discharge, sessions of rehabilitation, and progression of outcomes.
4. Outcomes:
   1. Primary outcomes:
      1. Daily activities: Barthel Index, instrumental activities of daily living.
      2. Frailty: Clinical Frailty Scale
   2. Secondary outcomes:
      1. Cognition: Short Portable Mental State Questionnaire
      2. Depression: Geriatric Depression Scale-5 Item
      3. Delirium: Confusion Assessment Method
      4. Fall risk: Stop Elderly Accidents, Deaths, and Injuries
      5. Potentially inappropriate medication: Beer’s criteria 2015
      6. Nutrition: mini Nutritional Assessment- Short Form
      7. Quality of lives: EuroQol-5 dimension
      8. 14-, 30-, 60-, and 90-day Readmission, and ER visits: times and reason

We will analyze the primary and secondary outcomes between the PAC groups and the control group. We will report the outcomes and differences between groups.

The SPSS (SAS) will be used for statistical analyses. Continuous variables were presented as mean ± standard deviation, and categorical variables were presented as percentages or times. The analysis of variance will be used for continuous variables to compare differences before and after PAC-frailty. The chi-square test will be used for categorical variables to compare differences before and after PAC-frailty. All p-value < .05 will be set as statistical significance using a two-tailed test.

Expected schedule after approval of IRB:

1. Literature review: the first month
2. Organization of medical record: the second month
3. Analysis: the third month
4. Reporting and submission: the fifth and sixth month

There could be some difficulties during our research program (i.e. incomplete data). This problem will be solved by statistical adjustments.

1. **Expected benefits**

We expect that after PAC-frailty, the outcomes will be improved including activities of daily living, frailty, acute delirium, nutrition, depression, cognition, medication, and fall risks. Also, the readmissions and ER visits will decrease.

| Gantt Chart | | | | | | | | | | | | | |
| --- | --- | --- | --- | --- | --- | --- | --- | --- | --- | --- | --- | --- | --- |
| Month  Work | 1 | 2 | 3 | 4 | 5 | 6 | 7 | 8 | 9 | 10 | 11 | 12 | Note |
| Seeking IRB approval |  |  |  |  |  |  |  |  |  |  |  |  |  |
| Review of literature |  |  |  |  |  |  |  |  |  |  |  |  |  |
| Data collection |  |  |  |  |  |  |  |  |  |  |  |  |  |
| Statistical analysis |  |  |  |  |  |  |  |  |  |  |  |  |  |
| Manuscript preparation and writing |  |  |  |  |  |  |  |  |  |  |  |  |  |
| Manuscript submission |  |  |  |  |  |  |  |  |  |  |  |  |  |
| Working schedule(%) | 10 | 20 | 25 | 30 | 40 | 45 | 50 | 60 | 70 | 80 | 90 | 100 |  |

1. **Expected schedule**
2. **References**
3. Department of Health. Integrated Post-Acute Care Project. June 26, 2017.
4. Miquel Angel Mas,Sergi Sabate, Marco Inzitari,Sebastia J Santaeugenia Gonzalez;Hospital-at-home Integrated Care Programme for the management of disabling health crises in older patients:Comparison with bed-based Intermediate Care; Age and Ageing 2017; 0: 1–7
5. American Geriatrics Society 2015 Beers Criteria Update Expert Panel; American Geriatrics Society 2015 Updated Beers Criteria for Potentially Inappropriate Medication Use in Older Adults; J Am Geriatr Soc 63:2227–2246, 2015
